# Computational Model-Based Analysis of Strategies to Enhance Scaffold Vascularization

**DOI:** 10.1089/biores.2016.0039

**Published:** 2016-11-01

**Authors:** Elif Seyma Bayrak, Banu Akar, Sami I. Somo, Chenlin Lu, Nan Xiao, Eric M. Brey, Ali Cinar

**Affiliations:** ^1^ Department of Chemical and Biological Engineering, Illinois Institute of Technology, Chicago, Illinois.; ^2^ Department of Biomedical Engineering, Illinois Institute of Technology, Chicago, Illinois.

**Keywords:** scaffold vascularization, agent-based modeling, strategies for enhancing vascularization

## Abstract

Stable and extensive blood vessel networks are required for cell function and survival in engineered tissues. A number of different strategies are currently being investigated to enhance biomaterial vascularization with screening primarily through extensive *in vitro* and *in vivo* experiments. In this article, we describe an agent-based model (ABM) developed to evaluate various strategies *in silico*, including design of optimal biomaterial structure, delivery of angiogenic factors, and application of prevascularized biomaterials. The model predictions are evaluated using experimental data. The ABM developed provides insight into different strategies currently applied for scaffold vascularization and will enable researchers to rapidly screen new hypotheses and explore alternative strategies for enhancing vascularization.

## Introduction

Cell-based approaches in tissue engineering require rapid and stable vascularization of biomaterials to regenerate tissues with high oxygen demand. However, clinical applications of cell-based approaches have been primarily limited to relatively avascular cartilage^1^ or thin skin.^2^ Well-developed vasculature is essential for almost all engineered tissues and is particularly challenging in thick tissues.^3^

Researchers have investigated a number of different strategies to establish vascular networks within biomaterials. These include: (i) optimizing design of physical and chemical scaffold architecture to enable or even enhance vessel growth, (ii) delivery of angiogenic factors to stimulate vessel growth from host tissue, and (iii) building vascular networks within the scaffold before implantation (*in vitro* prevascularization).^4,5^ For the first strategy, a broad range of physical properties of the scaffold can result in biomaterials that are more permissive for directed and rapid vessel invasion.^6–10^ Not only does pore size and porosity influence angiogenesis but also interconnectivity of the porous structure influences the rate of angiogenesis.^11^

Delivery of angiogenic factors to target microenvironments in scaffolds to stimulate cell migration and proliferation can enhance vascularization.^12–15^ A variety of angiogenic factors such as vascular endothelial growth factor (VEGF), basic fibroblast growth factor (bFGF), and/or platelet-derived growth factor (PDGF) have been delivered from scaffolds, aiming to enhance the vascularization process.^16–19^

However, the typical approach to deliver growth factors (GFs) from scaffolds does not allow precise control over spatiotemporal release and often results in inadequate vascularization. Spatial and temporal gradients of GF affect the direction, structure, rate of cell invasion, and vascularization.^20,21^

While promising results are being obtained using these approaches, the depth of vascularization may not be sufficient to reach the cells located in deeper parts of larger scaffolds. In addition, the newly formed capillaries induced by external GFs may be unstable and regress after the GF withdrawal.^22^ Prevascularization is another approach that interests tissue engineers to promote rapid vascularization.^23^ In this approach, a partial capillary network is established within an implantable scaffold before implantation,^24^ so that new blood vessels that grow from host vessels can make connections with these preexisting capillaries and establish blood flow more rapidly to deeper regions of the biomaterial. Studies have shown that preformed microvascular networks were successfully perfused after implantation and gave rise to functioning networks.^25,26^

Systematic evaluation of these approaches strictly through *in vivo* or *in vitro* experimentation is costly and a single experiment can take several months depending on the application. Moreover, understanding interactions of different components, explaining the causes for possible outcomes, and predicting future events may not be possible with experimentation alone. The need for computational modeling in tissue engineering field is therefore well recognized.^27^ Angiogenesis in tissue-engineered constructs has been simulated using various computational approaches to investigate the effects of mechanical stresses, exogenous delivery of an angiogenic GF,^28^ seeding the scaffold with vascular cells,^29^ and manipulating pore structure.^30,31^

Agent-based models (ABMs) have been used successfully in recent years for modeling biological systems.^32–34^ Unlike traditional modeling approaches, ABMs simulate the changes in the behavior of individual agents instead of, or in combination with, system level variables. Agents behave based on simple rules that originate from knowledge of the system under study. An ABM was developed in our group previously to simulate the effects of biomaterial pore structure on angiogenesis, focusing on the endothelial cell (EC) (the cells lining the walls of the blood vessels) behavior.^30,31^ This model has been modified and extended to study the effects of strategies, including GF release and prevascularization of biomaterials on angiogenesis. The results will focus on the investigation of the impact of: (i) scaffold architectural design, (ii) gradient and GF release, and (iii) prevascularization on scaffold vascularization and comparison of simulation results with relevant experimental studies.

## Materials and Methods

### ABM of angiogenesis and endothelial cell agent

**FIG. 1.**
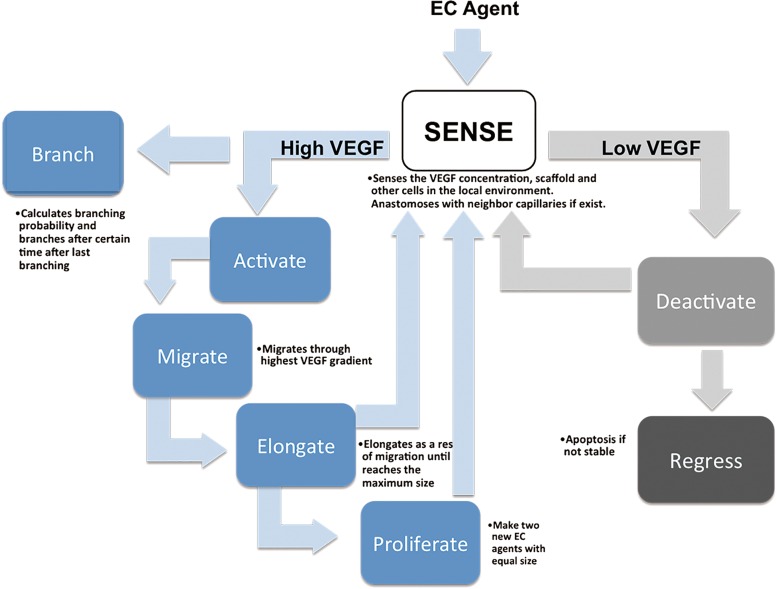
Abstracted rule-base governing the behavior of each endothelial cell agent.

The angiogenesis simulation model is constructed based on the actions of EC in response to the presence of soluble factors and the biomaterial microenvironment. EC behavior was abstracted into a rule-base (Fig. 1) based on knowledge of the real angiogenesis process observed in experimental studies from ourselves and others. ECs become activated when the GF concentration reaches a threshold in the local environment. The activated ECs (tip cells) migrate up the GF gradient. While the tip cell is migrating, stalk cells elongate until they reach a maximum size. Subsequent elongation results from the proliferation of new ECs (stalk cells). GFs are also needed for newly formed capillary survival since ECs undergo apoptosis in regions of GF deficiency resulting in capillary regression.^22^ A minimum threshold is used in the ABM to identify the point where capillary regression would occur. Anastomosis occurs when two elongating EC agents from different capillaries are within a defined distance from each other. The two sprouts will connect to form a stable blood vessel, which is assumed to allow blood flow; pressure gradient necessary for flow is not considered. ECs cannot invade nondegraded scaffolds. When an EC encounters part of a scaffold, it searches new routes to keep moving through the gradient. Parameters and detailed rule base governing the EC behavior are derived from the literature and have been reported elsewhere.^31^

### Model scaffolds

**FIG. 2.**
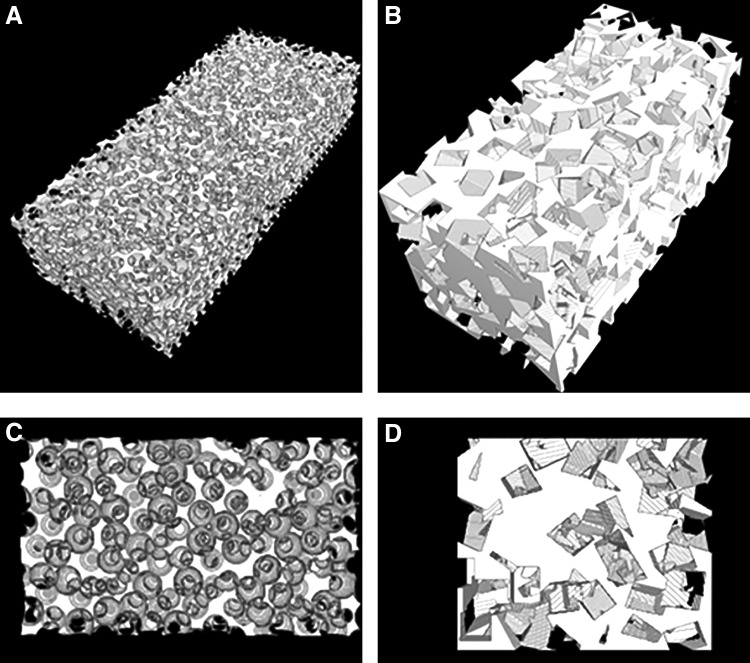
Images of the bulk scaffold structures **(A, B)** and interfaces **(C, D)** for spherical **(A, C)** and rectangular pores **(B, D)**.

Three-dimensional models of scaffolds with spherical and rectangular pores were generated computationally to study the effect of biomaterial pore interconnectivity on vascularization as described previously.^11^ The interconnectivity of the scaffolds is defined as the mean diameter of the opening between adjacent pores. Normalized pore connectivity (NPC) is calculated for each scaffold as the ratio of the mean pore interconnectivity to the mean pore diameter. The scaffolds were initiated by specifying a random pore location. Subsequent pore locations were generated by searching all locations within the scaffold that would satisfy the condition of the specified interconnectivity. Once the locations met the criteria, the pores were placed followed by, again, searching locations from displaced pores that would satisfy the interconnectivity criterion. These processes were repeated until no locations within the scaffold were able to satisfy the specified interconnectivity. For scaffolds with a constant pore size, porosity is dependent on the pore interconnectivity. Bulk and interface porosity of the lower (NPC 0.25) and the higher (NPC 0.45) pore throat diameter are reported. Three-dimensional renderings of spherical scaffolds with corresponding interface are shown in Figure 2A, C.

Scaffolds were also modeled with rectangular pores to approximate salt-leached scaffolds with the salt crystal size range consistent with experiments, 300–500 μm. Similar to spherical scaffolds, pore locations were determined randomly within a matrix. Subsequent pore locations were determined based on amount of overlap between neighboring pores. Once all locations within matrix were determined, rectangular pores were added to the matrix. Each rectangular pore was randomly rotated, fixed at its center, for the x, y, and z dimensions between 0° and 360° in intervals of 10°. These processes were repeated until a desired porosity was reached. In these experiments, we used 64.4%, which is based on experimental values of salt-leached hydrogels fabricated experimentally. Three-dimensional renderings of cylindrical scaffolds with corresponding interface are shown in Figure 2B, D.

### Growth factor release profiles

For gradient studies, the models of salt-leached scaffolds were used, and a gradient of an angiogenic GF, platelet derived growth factor-BB (PDGF-BB), was added to the structure.^20^ A layer of degradable hydrogel containing microspheres (distal layer) is placed on the surface of a porous fibrin/poly (ethylene glycol) (PEG) composite scaffold. PDGF-BB was encapsulated into degradable microspheres (poly (lactic-co-glycolic acid)) (PLGA) and microspheres and placed at the top of the scaffold within the degradable polymer. Scaffolds were kept in cylindrically shaped shells, and host vasculature was located at the bottom of the scaffold to approximate one-dimensional diffusion (y-direction). PDGF-BB gradients through the porous hydrogel system due to degradation of microspheres and diffusion in the hydrogel structure were modeled in MATLAB based on Fick's second law. In the model, diffusion is assumed as the only mechanism of transport. Boundary condition at the distal layer was adapted from the experimental release kinetics data, and the surrounding tissue was modeled as infinite sink PDGF-BB, which has a low affinity for fibrin within the porous structure.^35,36^ Hence, it was assumed that PDGF-BB did not interact with the scaffold. The effective diffusion coefficient within the scaffold was calculated by fitting the model to experimental results to account for both steric effects of the scaffold structure and any binding to fibrin. The experimental release findings and details of the diffusion model were reported previously^20^

**FIG. 3.**
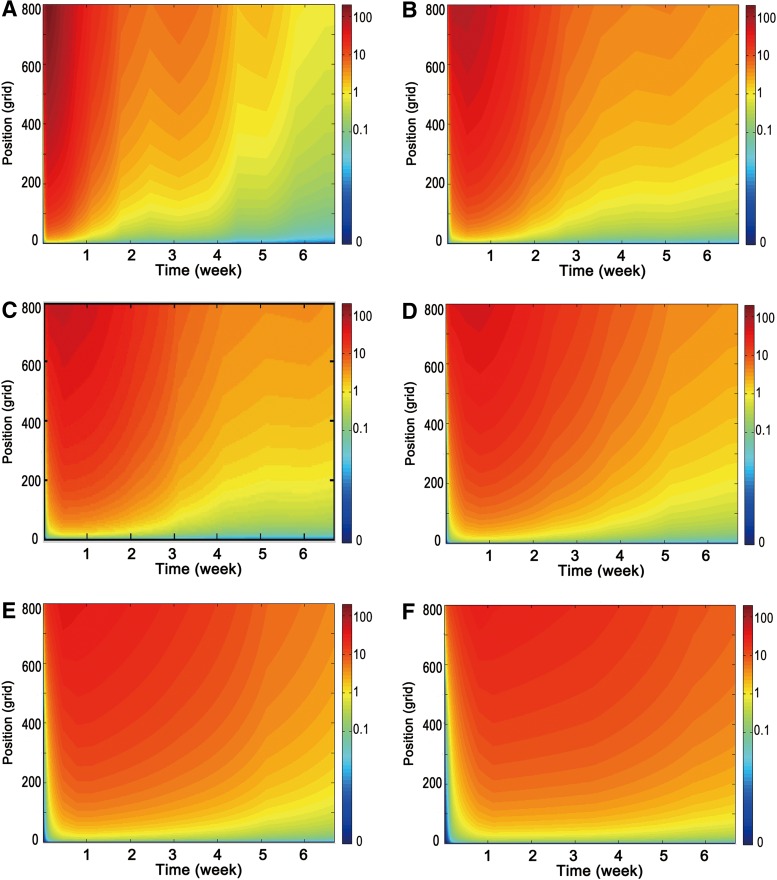
Different release speed of GF from distal layer of biomaterial. Concentration profile within **(A)** current experimental system (Case A), **(B)**%20 slower (case B), **(C)**%50 slower (case C), **(D)**%200 slower (case D), **(E)**%300 slower (case E), **(F)**%500 slower (case F). GF, growth factor.

Six different concentration cases were generated to run in the ABM to investigate the effects of varying release rates on angiogenesis. Case A indicates the concentration profile studied in the current experimental system. Cases B, C, D, E, and F indicate 20%, 50%, 200%, 300%, and 500% slower release rate than Case A (Fig. 3). To generate slower release conditions, diffusion profiles were expanded by increasing time ticks in the solver. The concentration data were then multiplied by a correction factor to keep the total PDGF-BB dose (200 ng) the same for all cases.

### Prevascularized scaffolds

Capillary segments are replaced into the porous media of modeled scaffolds to create *in silico* a prevascularized scaffold. EC agents are randomly located as initial source to start the capillary segments. ECs are continuously added to the end of capillary chain until the randomly selected location is not a part of the scaffold. If no pore location is found after several trials, the algorithm stops the capillary chain. These random stops cause a variation in capillary length within the scaffolds.

## Experimental Studies

### Preparation of gradient scaffolds

Porous gradient scaffolds were formed using either poly (methyl methacrylate) (PMMA) with a sphere templating method (for interconnectivity studies) or salt-leaching method for GF release studies. Details of the methods for scaffold preparation were explained previously.^20^ Briefly, PEG (250 mg/mL, Mw ∼8000) was dissolved in deionized (DI) water containing 0.5% w/v Irgacure 1173 (Sigma Aldrich, St. Louis, MO) to prepare hydrogel precursors for both methods. Thrombin (100 U/mL) (Sigma Aldrich, St. Louis, MO) was added to precursor for salt-leaching technique.

Templates using PMMA microspheres (106–125 μm diameter) (Cospheric, Santa Barbara, CA) were created to make hydrogels with varying NPC. The hydrogel precursor was poured over PMMA templates and polymerized under UV light (365 nm) for 5 min. The hydrogels were washed with acetone several times and incubated in acetone overnight to remove the PMMA microspheres. The hydrogels were then incubated in DI water for 2 days. The resulting hydrogels had a NPC of 0.24 and 0.42. For the salt-leached gels, 200 μL of hydrogel precursor was mixed with 400 mg sieved salt crystals (300–500 diameter) and polymerized under UV light (365 nm) for 5 min to generate hydrogels. Hydrogels were incubated in DI water overnight to leach out salt crystals.

To generate GF gradients within the scaffolds, PDGF-BB loaded PLGA microspheres were placed at the top of the hydrogels within a degradable hydrogel layer (poly (ethylene glycol L- lactic acid) (PEG-PLLA)). Different GF concentrations (1, 10, and 100 μg/mL) were encapsulated into PLGA microspheres and combined with scaffolds. Three hundred microliters of a fibrinogen solution (40 mg/mL in saline) was added dropwise into the pores of the hydrogel.^37^ Then, the gel was incubated at room temperature for 30 min to allow interaction of fibrinogen with thrombin to form the fibrin.

### *In vivo* testing

**FIG. 4.**
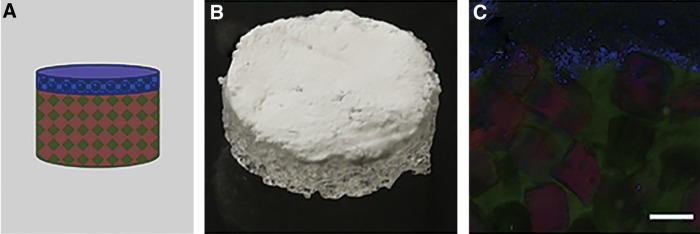
Formation of multilayer scaffold system. **(A)** Schematic representation of the gradient scaffold. **(B)** Gross image of the gradient scaffold. **(C)** Confocal image of a vertical section of the gradient scaffold indicating presence of the distal layer containing the PLGA microspheres (blue), underlying porous scaffold structure (green), and fibrin (red) loaded within the pores. Scale bar is 100 μm (with permission from [20]). PLGA, poly (lactic-co-glycolic acid).

Animal experiments were performed at Edward Hines, Jr. VA Hospital with procedures approved by the Institutional Animal Care and Use Committee. The scaffolds were placed in poly(propylene) shells that allowed orientation of the GF source distal to the surrounding tissue. The opposite end of the scaffold is open to the tissue below (Fig. 4). Sterile scaffolds with low (0.24) and high (0.42) interconnectivity, and salt-leached scaffolds with varying GF concentrations (1, 10, 100 μg/mL) were implanted into rodents using a subcutaneous implantation model.^20^ Implants were harvested at 3 and 6 weeks (*n* = 5 animals for each time point). Hematoxylin and Eosin stained sections were imaged using an Axiovert 200 inverted microscope (5× objective, 1.10 mm/pixel). Tissue invasion depth was measured as the straight-line distance from the host tissue to the deepest tissue location where tissue could be identified within the scaffold.

### Model evaluation

3D virtual scaffold is assumed to be sliced into three vertical pieces, and the results are averaged over these pieces and reported. Normalized vessel invasion is defined as the distance between host and the deepest part of the scaffold that blood vessels reached over total size of the scaffold in that direction. Normalized anastomosed vessel invasion is defined similarly; however, only blood vessels that were anastomosed and stable were taken into account for calculation. Total capillary length is calculated within the model considering the length of each EC contribution to the blood vessel network. Comparison with experimental data was performed assuming that tissue invasion is equivalent to blood vessel invasion in the model since tissue formation is not explicitly modeled in this study.

### Statistical analysis

Statistical analysis was conducted to compare simulation predictions with experimental results for high interconnectivity and high GF concentration cases. Paired Student's *t*-test was used for comparison of normalized invasion depth between different time points in simulation and experiment results, respectively. Unpaired Student's *t*-test was used for comparison of normalized invasion depth between simulation results and experimental data at different time points. Statistical data are expressed as mean ± standard deviation. Values of *p* < 0.05 were considered statistically significant.

## Results

### Effect of pore interconnectivity on vascularization

**FIG. 5.**
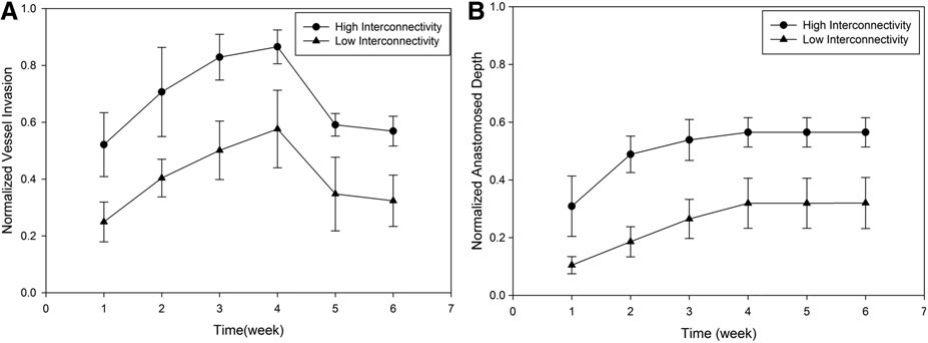
**(A)** Normalized vessel invasion and **(B)** normalized anastomosed vessel invasion depth for biomaterials with low and high interconnectivity.

Simulation results of vessel invasion and anastomosis depth in scaffolds with different interconnectivities indicate that high interconnectivity scaffolds have twice as much vessel invasion compared to low interconnectivity scaffolds (Fig. 5). Both high and low interconnectivity had increasing vessel invasion up to week 4. Due to decreasing GF levels after the fourth week, the invaded capillaries began to regress and a significant reduction in vessel invasion depth was observed. The results of anastomosed invasion depth were similar comparing to that of vessel invasion depth until week 4. However, the anastomosed invasion depth remained constant after that. This is because anastomosed invasion depth represents the invasion of stable vessels that are assumed to establish blood flow and not prone to regression. After blood flow is maintained, the vessels are treated as independent of GF levels. No additional sprouting occurred after 4 weeks due to the low GF concentration. However, capillary regression did not occur and the anastomosed invasion depth remained constant. In high interconnectivity scaffolds, around 50% of the scaffolds were vascularized by stable vessels by the fourth week, whereas this value is around 30% for lower interconnectivity scaffolds. The results demonstrated that pore interconnectivity has a significant impact on vessel invasion. Higher NPC scaffolds are more permissive for vessels to invade into deeper parts, whereas lower NPC scaffolds result in a significant spatial hindrance to vessel invasion.

**FIG. 6.**
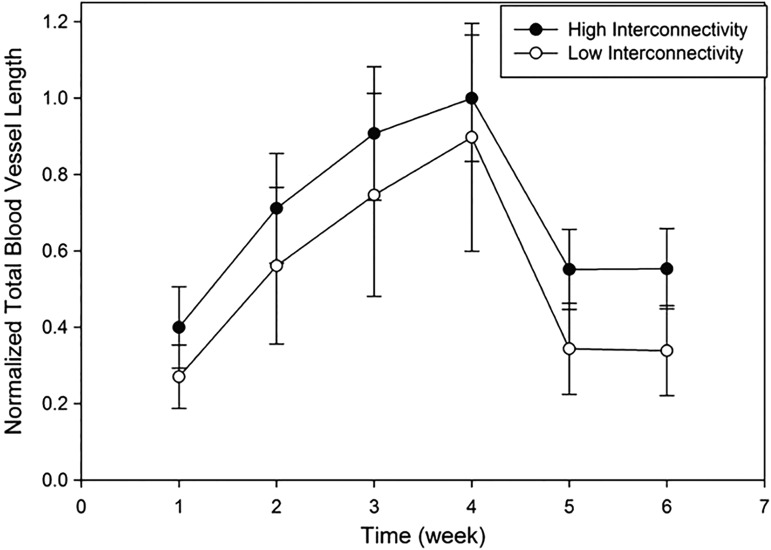
Total blood vessel length for biomaterials with low and high interconnectivity.

Total blood vessel length observed in both interconnectivities was not significantly different from each other (Fig. 6). This indicated that approximately the same number of capillaries formed in both scaffolds, and the lower interconnectivity scaffolds resulted with higher blood vessel density closer to interface.

**FIG. 7.**
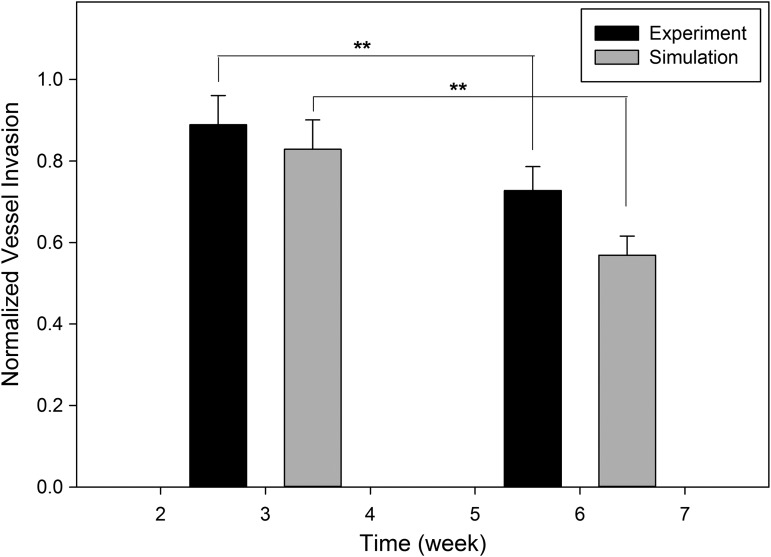
Comparison of simulation predictions with experimental results for high interconnectivity scaffolds. **p < 0.05.

Simulation results for vessel invasion depth were compared to experimental studies (Fig. 7). Simulation results follow the experimental findings in terms of both absolute value and trends. There was no significant difference (*p* > 0.05) in normalized vessel invasion depth at week 3 between the simulation results (0.8289 ± 0.0721) and experimental data (0.8888 ± 0.0719). Same conclusion can be made for week 6. Capillary regression was observed for both simulations and experiments from week 3 to 6, which resulted in significant decrease (*p* < 0.001) of normalized vessel invasion depth (0.7274 ± 0.0587 for experiments and 0.5689 ± 0.0470 for simulations).

### Gradient growth factor delivery

The ABM model was also used to investigate the effects of different total dose (2, 20, and 200 ng) of GF on vascularization within porous scaffolds. These total doses of GF correspond to 1, 10, and 100 μg/mL loading concentrations in experimental conditions, respectively. Scaffolds are assumed to have a distal layer that is composed of GF encapsulated microspheres within a degradable layer. The dynamic concentration gradient was generated based on known GF release kinetics from the degrading microspheres followed by diffusion through the porous polymer scaffolds. Simulations were performed within scaffolds (loaded with 1, 10, and 100 μg/mL of GF) for 6 weeks. Dimensions of the scaffolds used in this study were 4 mm height and 10 mm diameter, consistent with a typical experiment. Scaffold has a heterogeneous pore shape. The pore size of the scaffold varied between 300 and500 microns, and the porosity was 0.64 ± 0.64si.^20^ The interconnectivity was not controlled within the scaffold.

**FIG. 8.**
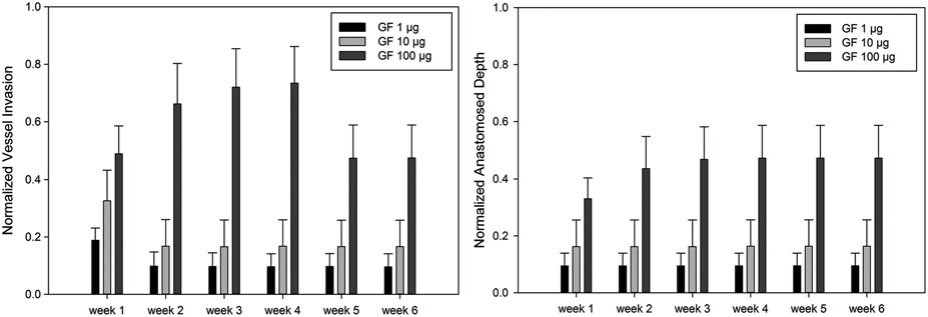
Normalized vessel and anastomosed vessel invasion depth with varying GF concentrations.

Figure 8 illustrates the effects of GF concentration on vessel invasion within the scaffold. The highest concentration (100 μg/mL) resulted in the deepest vessel invasion for all time points. Capillary regression was observed after the fourth week with 100 μg/mL case, whereas regression started immediately after the first week in the 10 and 1 μg/mL cases. No significant changes in vascularization behavior were observed after the first week in lower concentration gradients.

**FIG. 9.**
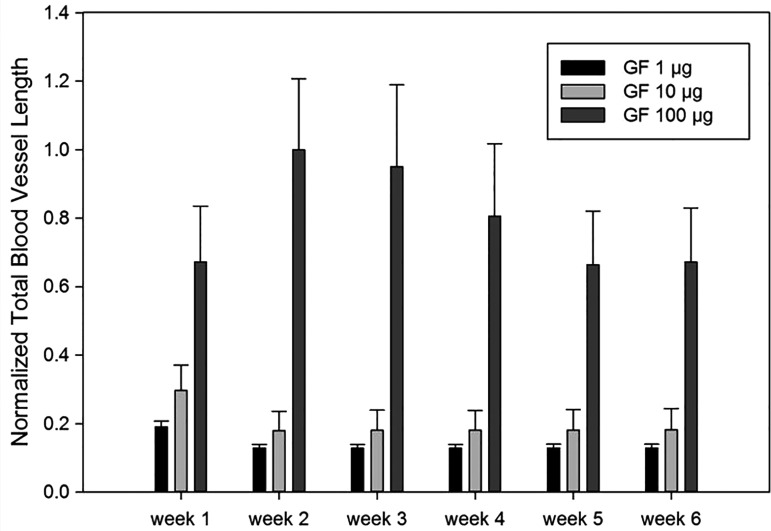
Simulated total blood vessel length with varying total dose of GF concentrations.

Different GF concentrations affected total blood vessel length as well (Fig. 9). The highest total blood vessel length was observed in the highest GF concentration. The slight reduction in capillary length began after the fourth week with the 100 μg/mL case, whereas it happened immediately after the first week with lower GF concentrations.

**FIG. 10.**
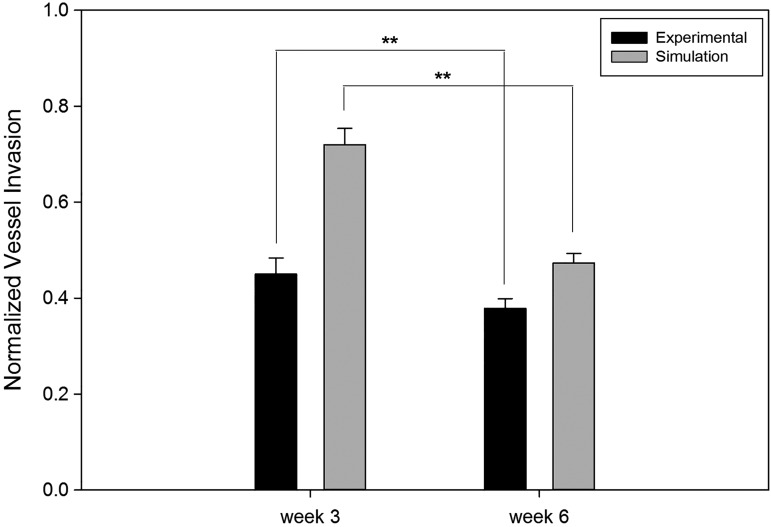
Comparison of simulation predictions with experimental results for 100 μg/mL GF loaded scaffolds. **p < 0.05.

The model results were compared with experimental results using PEG/fibrin composite hydrogels with increasing concentrations (0, 1, 10, and 100 μg/mL) of PDGF-BB. The comparison of simulation and experimental results for vessel invasion at the highest GF concentration (100 μg/mL) is presented in Figure 10. Simulation results showed good agreement with experimental studies and both suggest that GF gradients rapidly accelerated vascularization into the porous scaffolds. There was significant difference (*p* < 0.001) in normalized vessel invasion depth in week 3 between the simulation results (0.7202 ± 0.1342) and experimental data (0.4502 ± 0.0337). Capillary regression was observed for both simulations and experiments from week 3 to 6, which resulted in significant decrease (*p* < 0.001) of normalized vessel invasion depth (0.3788 ± 0.0200 for experiments and 0.4736 ± 0.1147 for simulations).

**FIG. 11.**
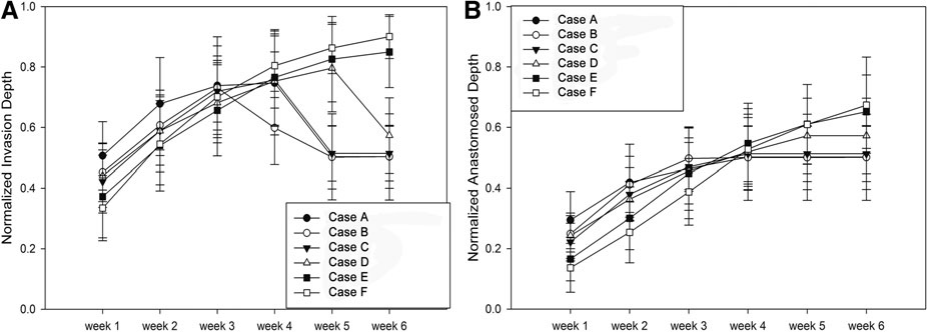
Normalized vessel **(A)** and anastomosed **(B)** invasion depth for delayed release conditions.

Even with the highest concentration case (100 μg/mL), blood vessel regression is observed due to GF reduction at later time points. Previous studies reported that the minimum GF concentration required for cell activation is in the order of 1 ng/mL.^36^ Since this threshold value was implemented into ABM, blood vessel regression period was successfully predicted within scaffolds, which mimic the current experimental system. Furthermore, we have investigated varying release rates within the system to eliminate/decrease vessel regression. The diffusion model was modified to generate concentration profiles with altered release kinetics compared to the currently investigated experimental scaffold. Simulation results of vessel invasion and anastomosis depth for computationally generated slower growth release cases (B, C, D, E, F), but at the same total dose (100 μg/mL), are illustrated. (Fig. 11).

For the control case, vessel invasion depth reached its highest value at around the fourth week and then started to regress. For the 20% (Case B) and 50% (Case C), slower release rate did not significantly affect the regression point. However, 200% slower release resulted in regression occurring after 5 weeks. The regression time was postponed if we decreased GF release rate, and there is no vessel regression in 300% slower (Case E) and 500% slower (Case F) even after sixth week. The initial vessel invasion depth in the slower GF release cases is slightly smaller compared to the control case due to the decreased burst release in the early time points. However, blood vessel regression was limited in these cases due to the sustained release of GF, which eventually resulted in a larger invasion depth. The results showed that capillary growth and regression varied significantly with the different release rate of GF and that sustained release promotes scaffold vascularization.

### Prevascularized scaffolds

**FIG. 12.**
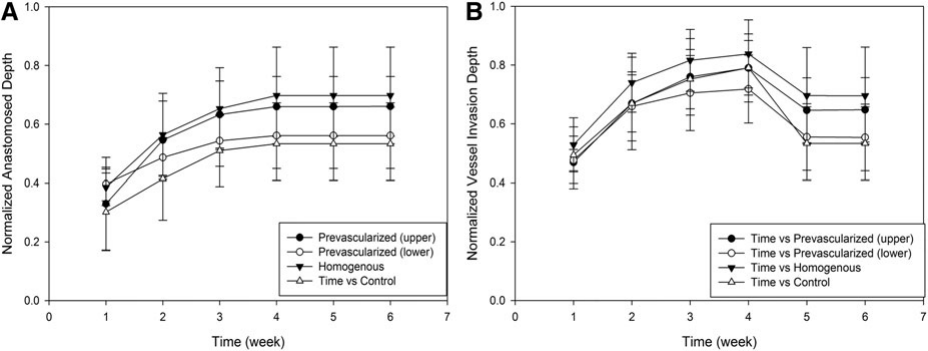
Normalized anastomosed vessel **(A)** and vessel invasion depth **(B)** for prevascularized scaffolds.

**FIG. 13.**
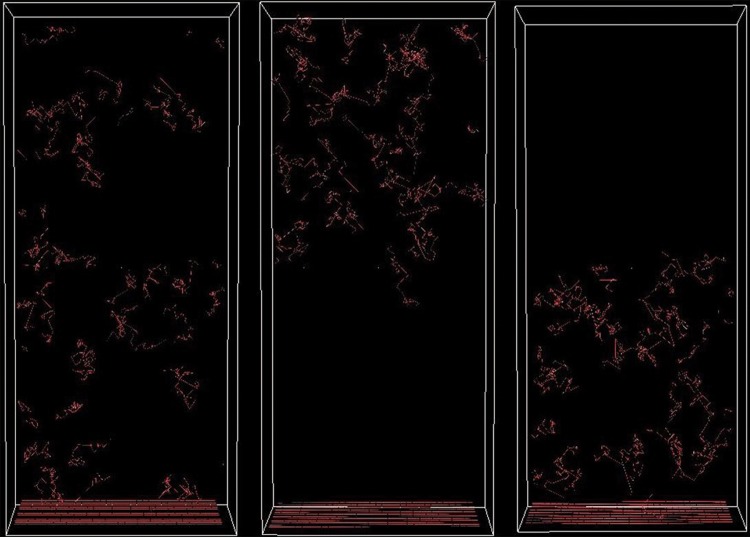
Simulation interface for homogeneous (left), top half (middle), and lower half (right) prevascularized scaffolds.

Prevascularized scaffolds can be formed *in vitro* from seeded ECs before *in vivo* implantation.^38^ In this article, we have simulated angiogenesis after the implantation of prevascularized scaffolds. Three configurations of prevascularized biomaterials were generated (Fig. 12). In the first case, the entire scaffold medium was filled with capillary segments. In the second and third cases, either the bottom or top half part of scaffolds in y-dimension was prevascularized, respectively. Vascularization was investigated in all structures, and results were compared with a control case where there were no preformed capillaries (Fig. 13). Simulation runs were performed under high GF (100 μg/mL) concentration and scaffolds with higher interconnectivity.

Entirely prevascularized (homogeneous) scaffolds showed highest and fastest final vessel invasion. Top-half prevascularized structures showed slightly lower and initially slower vessel invasion compared to homogeneous case. Bottom-half prevascularized case resulted in high initial invasion, but after second week it was only slightly better than the control case. Over 50% of the scaffold was vascularized with anastomosed vessels in top and entirely prevascularized cases within the first 2 weeks, whereas in the control and lower prevascularized cases, this took 6 weeks. The connection between the preformed vessels and the capillaries from the host enabled more blood flow circulation through the vessels, resulting in stable vessels that resist regression. Regression was reduced significantly after fourth week in top and entirely prevascularized scaffolds.

## Discussion

The aim of this study was to develop a computational framework for studying the combined effects of scaffold design, GF release kinetics, and use of preformed capillaries on biomaterial vascularization. Simulation results were compared with experimental findings. Both simulation runs and experimental studies showed that properly designed scaffolds can lead to well-vascularized scaffolds. Vascularization is greatly influenced by pore interconnectivity, with smaller openings between pores hindering blood vessel invasion into the scaffolds.

Controlled GF release is a common approach to engineer tissues. Many *in vivo* experiments have shown that GFs can enhance scaffold vascularization in a variety of ways.^39–42^ We analyzed both the effect of magnitude and release kinetics of GFs on scaffold vascularization. The invasion depth was influenced by the total dose. Higher concentration (100 μg/mL) resulted in greater vessel invasion compared to lower doses (1 and 10 μg/mL). Even with the highest concentration case (100 μg/mL), endothelial capillary regression is observed due to the GF reduction at later time points. Furthermore, we investigated the role of release rates within the system on vascularization. Scaffolds were generated with GFs at the same dose, but slower release kinetics. The comparison between control case (matched to experimental conditions) and slower release cases demonstrates that sustained slower release can promote more persistent vessel networks, deeper vessel invasion, longer total blood vessel length, and larger anastomosed depth. Although diffusion profiles were created computationally, these results can guide experimental studies. GF release can be varied by modifying degradable layer of the scaffold in future experiments.

Even though scaffolds with high interconnectivity, higher GF dose, and sustained GF release showed better vascularization results, the rate of vascularization was still limited relative to engineering large volume tissues. At the end of 6 weeks, only ∼50% of the scaffold was vascularized. In these systems, vascularization depends on mainly GF concentration, physical structure of the scaffold, and host vasculature. However, the presence of preformed networks within the scaffold may enhance vascularization. The prevascularized scaffold strategy was added to the model. The presence of preformed capillary segments significantly improved the rate of vascularization. Over 50% of the scaffold was vascularized within the first 2 weeks, and almost 80% of scaffold was vascularized by the end of the sixth week. The results were consistent with the finding from the literature that reported that preformed capillaries were connected to the host microvasculature and established of blood perfusion significantly faster compared with controls.^43^ ABM is also used to simulate experimentally not tested cases such as top and bottom only prevascularized scaffolds and suggested that when the top half is prevascularized, final vessel invasion can reach to deeper parts in the scaffold.

## Conclusion

Success in tissue regeneration is inherently linked with successful vascularization. The model developed in this study allows researchers to screen various hypotheses rapidly and explore alternative strategies to enhance vascularization. Simulation results were supported with experimental findings and suggested optimal designs and conditions for vascularization of engineered biomaterials.

## Abbreviations Used

ABM: agent-based model
bFGF: basic fibroblast growth factor
DI: deionized
EC: endothelial cell
GF: growth factor
NPC: normalized pore connectivity
PDGF: platelet-derived growth factor
PDGF-BB: platelet derived growth factor-BB
PEG: poly (ethylene glycol)
PLGA: poly (lactic-co-glycolic acid)
PMMA: poly (methyl methacrylate)
VEGF: vascular endothelial growth factor
